# “Breast is best”… until they say so

**DOI:** 10.3389/fsoc.2023.1022614

**Published:** 2023-03-09

**Authors:** Cristina Quinones

**Affiliations:** Department of People and Organisations, Faculty of Business and Law, The Open University, Milton Keynes, United Kingdom

**Keywords:** autoethnography, breastfeeding, standardized health, first-time mothers, identity work, intensive motherhood, feminism

## Abstract

In this autoethnographic article, I discuss the consequences of being exposed to two competing breastfeeding discourses during my first mothering experience—the “self-regulated dyad” and the “externally regulated dyad” discourse. The former represents the ideal scenario and the evidence-based practices recommended by the World Health Organization (i.e., breastfeeding on demand, internally regulated by the dyad). The externally regulated discourse refers to the standardized health interventions that take over when difficulties arise (e.g., weight gain deviations and latching issues). Building on Kugelmann's critique about our blind reliance on “standardized health,” existing evidence, and my breastfeeding journey, I argue that unqualified and unindividualized breastfeeding interventions are highly counterproductive. To illustrate these points, I discuss the implications of the polarized interpretation of pain and the limited dyadically focused support. I then move on to analyze how ambivalent social positioning around breastfeeding impacts our experience. In particular, I found that I was highly regarded as a “good, responsible mum” up till my baby was 6 months, and how breastfeeding became increasingly challenged by others when my daughter was approaching her first birthday. Here, I discuss how performing attachment mothering identity work allowed me to navigate these challenges. Against this backdrop, I reflect upon feminist ambivalent positionings on breastfeeding and the complexity of balancing the promotion of women's hard-earned rights while supporting them to engage in whatever baby-feeding choice they feel appropriate. I conclude that unless we acknowledge the physical and social complexities of the process, and our healthcare systems seriously invest in allocating human resources and training them appropriately, breastfeeding rates may continue to suffer and women continue to interiorize it as their own failure.

## 1. Introduction

The Global Breastfeeding Collective led by the World Health Organization (WHO) and UNICEF strongly recommends exclusive breastfeeding during the first 6 months of the baby's life and combining it with solids until the baby is 2 years old or more, as long as the mum and baby wish to do so (WHO, 2016; GPC, 2017; UNICEF and WHO, 2019). These recommendations stem from the accumulated evidence about the positive impact of breastfeeding on a baby's healthy development. Babies who do not breastfeed seem to experience more illnesses, worse prognoses, and longer-lasting conditions, and these effects do not only refer to the breastfeeding period but also refer to many years after that. Breastfed children “seem to perform better on intelligence tests and be less likely to be overweight and less prone to diabetes when they grow up” (WHO, 2023). For mothers, evidence suggests that breastfeeding reduces the risk of diabetes, breast and ovarian cancers, heart disease, and postpartum depression (Ip et al., 2007; WHO, 2016; UNICEF and WHO, 2019; Feltner et al., 2021).

The Global Breastfeeding Collective has set the ambitious goal of a 70% exclusive breastfeeding rate in the 0- to 6-month period after birth by 2030 (UNICEF and WHO, 2019). However, the worldwide breastfeeding rate is estimated at ~38%, and it continues to decline in many Western countries (Alianmoghaddam et al., 2017). Iceland is one of the countries with the highest breastfeeding rates, with 74% of women still breastfeeding at 6 months after giving birth and 27% at 1 year after giving birth (Símonardóttir, 2016). By contrast, the United Kingdom has one of the lowest figures in the world, the latest countrywide survey in 2010 revealed that only 1% of babies were exclusively breastfed at 6 months (Srivastava et al., 2020). Although there is no up-to-date figure about this period, a recent survey showed that just 48% of British women continue breastfeeding beyond 6–8 weeks (UKHSA, 2021). In Spain, 28.53% of women reported exclusive breastfeeding during the first 6 months and a further 18.42% used complementary feeding (i.e., breastfeeding supplemented by formula), adding up to a total of 46.5% (INE, 2021).

Considering the strong public health interest in exclusive breastfeeding, scholars have explored the obstacles explaining the low uptake. In a study conducted by Gianni et al. (2019), a total of 70.3% of mothers reported breastfeeding difficulties. Having cracked nipples was the most frequent barrier to breastfeeding 3 months after delivery (41%), shortly followed by the perception of insufficient milk (35.8%) and pain (31.2%). Similarly, Odom et al. (2013) found that 60% of women did not breastfeed as long as they intended to because of latching difficulties, pain, and baby weight gain concerns. Moreover, Bergmann et al. (2014) found that up to 80% of the mother–infant dyads reported difficulties. They also observed that a lack of appropriate healthcare and community support accounted for early abandonment. Unsurprisingly, the COVID-19 pandemic has worsened this situation. A study conducted with British mothers giving birth in February 2021 revealed that the percentage of women breastfeeding during the first few days went down compared to previous years and so did the immediate and long-term support received from healthcare professionals (IPSOS, 2022). The lower quality support during the pandemic has also been identified in Spain, a country with a similar public healthcare system (Muñoz-Amat et al., 2021; Rodríguez-Gallego et al., 2022). Taken together, this evidence suggests that pain, uncertainty about the baby's weight gain, and a lack of qualified support are critical factors in understanding both the negative experiences women face and the subsequent dropout.

### 1.1. Promoting breastfeeding

Despite how frequent the difficulties are, breastfeeding promotion and education campaigns seem to turn a blind eye to them and focus on the glossier side. In a large survey of British mothers who had planned to breastfeed their babies, most women reported having a great understanding of the benefits. However, respondents wished they had more information about the complexities of the process itself and argue that this would have encouraged them to breastfeed for a longer period (Brown, 2016). Importantly, unfulfilled intentions to breastfeed can have serious consequences. Borra et al. (2015) found that women who had intentions to breastfeed during pregnancy and were unable to do so afterward were at the highest risk of postnatal depression; whereas those who intended and were able to breastfeed had the lowest risk.

Taking my experience as an example, I illustrate how a more diverse representation of the challenges and experiences women go through when breastfeeding is needed. First, it can help new mothers develop more realistic expectations about the process, recognize themselves in some of these experiences, and be better prepared to decide on whether they wish to continue. Second, an acknowledgment of the complexities that breastfeeding involves challenges the “natural,” ergo “easy,” bias around breastfeeding and makes an explicit call for investment in qualified support (Bergmann et al., 2014).

### 1.2. Supporting breastfeeding

Breastfeeding is a physiological and inherently dyadic process that requires baby stimulation of the mother's nipple to produce milk. According to the WHO, breastfeeding works “on demand,” that is, as often as the babies want, day and night, as they soothe themselves and learn to satisfy their feeding needs (WHO, 2023). The WHO also recommends to not use pacifiers or bottles, as these require different sucking strategies and can interfere with breastfeeding. They also recommend not using formula supplementation unless there are clear medical reasons to do so. This is because formula decreases breastfeeding duration, and even when combined with breastfeeding, it reduces the benefits associated with exclusive breastfeeding (Kramer and Kakuma, 2012; Walker, 2015). Worryingly, studies have found that in-hospital breastfeeding interventions are often inconsistent, and formula supplementation is more frequently attributed to poor lactation management support than an authentic medical need (Cross-Barnet et al., 2012; Chantry et al., 2014; Bookhart et al., 2022).

In view of this, I argue that new mothers are exposed to the tensions of two competing discourses, each defining and prioritizing certain practices over others, and legitimizing ways in which breastfeeding can be supported—the “self-regulated dyad” and the “externally regulated dyad.” The former represents our current evidence-based understanding of breastfeeding, and it is the dominant discourse in the ideal case scenario (i.e., weight gain goes at the right pace and the mother does not report or experience any difficulty). By contrast, the externally regulated discourse refers to the medical practices and interventions that are carried out when difficulties arise to ensure the baby's health. As discussed earlier, these are neither always needed nor conducive to long-term breastfeeding success.

Building on Kugelmann (2003) critique about our blind reliance on “standardized health,” existing evidence, and my journey, I argue that unqualified, unindividualized breastfeeding support is indeed highly counterproductive and problematic. According to Kugelmann, standardized health is the quality that medicine aims to produce, has power over, and intervenes with its multiple technologies. Since the human body is the area of expertise of medical professionals, they are legitimized to define healthiness and, in turn, dictate behaviors and attitudes patients ought to have to achieve healthiness. While recognizing standardized health effectiveness and how it potentially provides the opportunity for health to the whole population, it also creates inequalities. The more we rely on standardized health to understand every single thing that happens to our bodies, the less in touch we are with how we feel and experience pain, wellness, and illness, and the less we draw on individual and community resources to help us heal. This sacrifice, according to Kugelmann, illustrates the counterproductivity of standardized health.

In standard healthcare interventions, like surgery or scans, the patient relies almost completely on the knowledge of the medical professionals. In breastfeeding, the “patient” holds a privileged knowledge, as breastfeeding is an embodied act regulated by the mum–baby dyad (Stearns, 2013). In this article, I discuss how interventions that ignore this basic understanding are indeed counterproductive, and they threaten not only breastfeeding success but also mothers' mental health.

Beyond medical understandings and practices, breastfeeding experiences are also shaped by the wider cultural discourses and norms, and western society has demonstrated a rather ambivalent position around breastfeeding (Earle, 2003). On the one hand, the “breast is best” slogan has inevitable moral connotations that shape our social constructions of “good mothering.” On the other hand, society is far from accepting the realities of “on demand” breastfeeding, including its public display. Thus, various studies have shown how mothers have to manage the tensions between breastfeeding and the comfort of others (Earle, 2003; Leeming et al., 2013; Sheehan et al., 2019). In this article, I illustrate the social ambivalence I experienced when I decided to continue breastfeeding beyond my baby's first year of life, and how identity work helped me navigate these challenges.

## 2. Methods

As a new mum, I lived and witnessed the consequences that strong breastfeeding campaigns coupled with poor understanding and allocated healthcare resources had on women around me and myself. Autoethnography is a research strategy that allowed me to use this lived experience to expose some of the perverse dynamics women face when navigating the complexities of this morally charged territory. As Hendrix (2020) states,

Autoethnography allows a researcher to show some aspect of his or her life within a particular context deemed as capable of shedding light on and, thereby, prompting a better understanding of some dimension of the human experience (p. 379).

Reflecting on my data, comparing and contrasting it with existing evidence, and assisted by the lens of my theoretical framework, I aim to unravel the effect that these contradictory discourses around baby feeding can have on first-time mothers, at a vulnerable time when our bodies ache and our previous identities take a back seat temporarily, to make space for motherhood (Sjöberg, 2019).

I used autoethnographic vignettes to report my personal experience. These have been used before in topics such as career journey (Humphreys, 2005), human resource management (Learmonth and Humphreys, 2016), and pain and disability (Esposito, 2014). In Humphreys' words, it helps me “to construct a window” (p. 845) to show the reader how living through the conflicting discourses around breastfeeding was having an impact on my confidence and mothering identity.

The vignettes correspond primarily to the first 6 months of breastfeeding my first daughter, during the first two waves of the COVID-19 pandemic, between June 2020 and January 2021. Most of the vignettes illustrate experiences I had during my hospital stay. I gave birth at the main University Hospital of Asturias, a northern Spain region. Although we were living in a small village, we had easy and close access to both the main hospital and local surgeries. The personal experience is analyzed in terms of the facts and emotions I experienced as a result of the tensions between the self-regulated vs. externally regulated discourse, the implications for the level of support I received, and how identity work and feminist positionings helped me navigate these challenges.

One of the key challenges of autoethnography storytelling is that its cathartic effects may get in the way of theory building (Griffin and Griffin, 2019). To prevent this, Griffin and Griffin argue that one must keep in mind the “theory which builds upon, and it is linked back to social theory”. I believe one of the ways that allowed me to move beyond catharsis was the time and experiences I had between writing these vignettes and writing this article. I began writing these vignettes when my baby was 1 year old. I felt exposed and digging on bounds that I would rather have left behind, as I was just starting to feel more like myself. According to Jones et al. (2016), this level of exposure is the sacrifice that autoethnography must do: “they need to be ready to risk their intimate and professional image through their research” (2015, p. 64). As I had started my healing process, I left the vignettes in a drawer and began writing the article again when my baby was about 2 years old.

During the time that passed between writing my vignettes and writing up the article, I ran several first-time mothers' support group sessions, exploring the issue of guilt in motherhood. This experience allowed me to further appreciate how we were collectively exposed to medical and social discourses that ultimately eroded the confidence we needed to craft our paths. I argue that this collective awareness and the time passed between writing the vignettes and going back to the article helped me gain a better perspective on my data, and further convinced me of the value of this research strategy that according to Griffin and Griffin (2019) “qualitatively and reflexively attempts to make sense of the self and society in an increasingly uncertain and precarious world.” On a broader professional level, the transition from a positivistic background to social constructionism I had begun years ago was finally validated by the greater understanding I reached through reflecting and grasping the concept of embodiment through my data. As Yoo (2020), an academic who also came out of the objective science constraints, rightly put it, this autoethnographic storytelling allowed me “to come out of the academic closet […] embracing the embodied ways of encountering the world and identifying myself with my hands through exploring” (p. 259).

## 3. Discussion

In this section, I discuss the impact that different societal and medical breastfeeding expectations and discourses, contradictory between and within themselves, had on me through a selection of vignettes about my breastfeeding experience around two normative stages (0–6 months and 6–12 months and beyond).

In Table 1, I present an overview of the dominant discourses at the aforementioned normative stages. The table includes examples of the breastfeeding norms according to each discourse, along with examples of potential scenarios of day-to-day breastfeeding, as well as the self and social scrutiny women may be subject to in those scenarios. The latter represents a compilation of some of the messages I received, read about, or other mothers shared with me during our breastfeeding journey.

**Table 1:** Breastfeeding as the initiation act to the guilt-shame mothering spiral.

**Baby's age**	**Breastfeeding discourse**	**Standard/norm**	**Scenarios**	**Mothering under self and social scrutiny**	**Emotions**
	Self-regulated dyad	Allow the dyad to connect as soon as the baby comes out of the womb, and the process works by itself	Pain and difficulties may arise, for instance baby doesn't take, sore nipples, mastitis, frenum surgery…	Why can't I do this? Why is it so painful? Why don't I fill her up? Is there something wrong with my (mothering) body?	Insecurity Guilt Shame
		Babies need to be fed “on demand”, whenever they want as much as they need			
**0-3 months**	Externally-regulated dyad	Baby has to put on “X grams” every week.. You should produce “X ml of milk” or …(formula supplement)	Baby doesn't gain the exact amount of weight required	External assessment of your breastfeeding skills… Are you putting his chick exactly what it has to be? Is her sucking functional? Does she have a frenulum?	Insecurity Guilt Shame
		You should offer on demand (current evidence-based advice)	If you offer on demand…	She might get used to asking too much…your baby is becoming breast-obsessed	
		VS.			
		You should offer “X times” a day (heritage from prior evidence-based advice)	If you offer “x times” a day…	Are you sure she is having what she needs? Is your baby under-nurtured?	
	Self-regulated dyad	You should continue offering on demand, and offer solids after the food	If the baby doesn't eat much one day…	Am I giving her too much milk? Shouldn't she be eating fruits by now?	Insecurity Guilt Shame
**6-12 months**	Externally-regulated dyad	Milk is the most important nutrient according to WHO	Baby eats solids nicely…	Is she taking the milk (and therefore the immunity) she needs?	
		VS.			
		You should start reducing dependence on breast, food takes priority (heritage from prior medical advice)	Baby does not eat much…	Am I creating a “breast addict”? Does she “boss me” around?	
	Self-regulated dyad	You should continue offering on demand, she will “wean off” when she is ready	Baby's feeding becomes more erratic and intense	Does she boss me around? Do I look like a hippie mum with my boob out all day?	Insecurity Guilt Shame
			Baby's wean off by themselves	What if my baby doesn't want to wean off? Have I made a “breast addict” baby? Was this all worth it if we are not in an underdeveloped country?	
**12-24 months**	Externally-regulated dyad	Breastmilk is recommended until 2 years old according to WHO	(as above)	(as above)	
		VS.			
		Baby does not need your milk anymore unless you live in an underdeveloped country	Stop breastfeeding	Other women around me still breastfeed, WHO recommends up to 2 years and beyond as long as baby and mum wish to do so…have I stopped too soon?	

### 3.1. The 0- to 6-month period

#### 3.1.1. About the pain

Public health campaigns advocating breastfeeding have contributed to equating “natural” with “easy,” free-flowing, and something that happens by itself (Bergmann et al., 2014). The fact that approximately two-thirds of lactating mothers experience physical difficulties tells us otherwise (Odom et al., 2013; Gianni et al., 2019). This under-represented side of breastfeeding in the promotion of education campaigns hits women in the midst of the complex postpartum period, revealing a huge discrepancy between idealized breastfeeding and the realities of breastfeeding “on demand.” Hence, it is unsurprising that we interpret the early difficulties as signs of “body error,” removing us from the self-regulated scenario and deepening our need for professional help. Unfortunately, as I describe later, support does not always come as consistent or as aligned with either the extensive breastfeeding readings I did during my pregnancy or even the WHO's recommendations.

*My baby girl was born at 2.32 in the morning. As soon as she saw the light, she crawled up to my nipple and began sucking. I felt so relieved that we managed to get the skin on skin right, as I read this was critical for breastfeeding success. After that, we were taken to the room and as I was so desperately crazy to get it right from the scratch, I asked one of the nurses to come and help me breastfeed and tell me if I was doing it right. This was probably around 5:00 am in the morning. She told me not to worry, as babies can be up to 5 h without feeding once they came out of the womb, she told me to rest and get some help from the morning shift. It didn't seem right and I didn't want to rest, so I kept trying, but it was terribly painful, and I knew there was something wrong. The morning after, when I asked for help, the morning nurse told me off for not challenging her colleague's advice, as if by being a mum, I should have known better*.

The urgency of the morning staff was completely different from the laidback approach of the night nurse. Similar differences have been reported in other studies, where night staff has prioritized mother's rest over breastfeeding support (Hauck et al., 2011; Schmied et al., 2011; Nickel et al., 2013). For me, this incident marked my (obsessive) quest to find common ground among the views of every single healthcare professional that came into my room. All the reading and preparation I had done did not prepare me for this lack of consistent advice from the people I thought were qualified to help me out. Instead, what I found were some very polarized responses; “*If it hurts, you are not doing it right”* vs. “*If it hurts, you just have to get used to it, like we all did.”* I even heard both viewpoints from the same person on different occasions. My nipples did hurt and bled, hence by some nurses' standards, I was not doing it right, while for others, I just had to get used to it. Unfortunately, the lack of consistent advice from healthcare professionals and midwives is commonplace, and one of the contributors to early cessation, particularly so, for primiparous women (Hauck et al., 2011). I was lucky to at least find some level of reassurance remembering my sister's experience, who managed to breastfeed her baby despite experiencing awful pain.

One of my lowest moments during the hospital stay was the time when one of the nurses, who believed the pain was something to get used to, took her responsibility a step too far.

*My partner was not allowed to be in the hospital any longer due to COVID-19 restrictions and at this point I already had very sore nipples. Part of me thought, you are doing ok, she is eating on demand, but the pain I was still experiencing really made me question my technique again and again. I asked one more nurse to help me. She pulled my boob so much that really hurt me, and ask me to just take it as it would get easier in future. I couldn't find energy to tell her off, as I would have liked to. My partner would have definitely do so, but he was not allowed there so he didn't see it*. *I couldn't hold my tears in, I felt terribly helpless, like a child looking after a baby. She suddenly saw me and soften her ways, she then decided to share her own experience of pain and the success that came after. As if she was telling me “I am doing it for your own good, hang on in there.”*

Breastfeeding promotion and education campaigns ignore the high prevalence of pain women experience or if they do, they regard pain as a sign of problems with latching or baby positioning (Brown, 2016; Símonardóttir, 2016). Importantly, pain is not only limited to the latching and early stages period but arguably, is a common feature throughout, even during extended breastfeeding. Thus, women report experiencing pain, for example, during the teething stages or as babies liberate the nipple when they fall asleep (Bunik, 2017).

I argue that the lack of acknowledgment of pain is problematic for various reasons. First, it infantilizes women, as it portrays what their bodies ought to feel when they engage in such a radically embodied event. Second, it emphasizes the romantic, beautiful side of breastfeeding, and ignores the difficulties, increasing the gap between the idealized experience and the lived experience for many, which ultimately erodes women's confidence and threatens their motherhood identity. Thus, it was precisely the discrepancy between the pain they experience during feeds and their beliefs in trouble-free breastfeeding that caused women greater distress and threatened their emerging mothering identities in Williamson et al. (2012) study. Ignoring or attributing one-sided interpretations of pain also contributes to exacerbating and individualizing the blame when women fail to meet those idealized standards. Thus, public health campaigns must provide a more balanced representation of pain. It is also important that we overcome the dichotomy between “get used to it” and “sign of error” and regard either as one possible explanation, as we showcase and validate the broader range of experiences women have. I argue that this may help women make fairer assessments of their success and better-informed decisions on whether they wish to continue.

#### 3.1.2. Support the dyad

In addition to the misrepresentation of pain, public health campaigns seem to be unilaterally focused on the importance of breastmilk to enhance the baby's wellbeing (Símonardóttir and Gíslason, 2018). Breastfeeding, however, is a dyadic process, a dyad that requires time and space to learn about and connect with their bodies and each other (Lau, 2018). The lactating mother and the baby rely on each other not only for the actual feeding but also for the required frequent stimulation to ensure that the breast milk supply is sufficient. For me, this connection was the most radical bodily experience I have ever had (besides giving birth). From the indescribable tingling sensation in my breasts when I was not near her and she began crying, to spontaneous milk spotting right until the end of our breastfeeding journey, just before she turned 2 years old.

*The first two months were exhausting, I was in pain, I had some episodes of mastitis and in addition to breastfeeding on demand, which effectively was constantly, I was asked to express milk just in case my baby was not taking all that she needed from directly breastfeeding due to her lingual frenulum. This meant that I was pretty much, all day, on breast call. During those days there were moments where my head (and my partner) were telling me to leave the house for a couple of hours. But I just couldn't do it, I felt a terrible animal urge to be there with her all the time, just in case, as if my mammal body was taking over, I couldn't do anything to resist*.

I argue that both the downplay of breastfeeding difficulties, and a unilateral focus on what is “best for the baby” ignores the inherently interactive nature of breastfeeding and reduces it to a disembodied transaction where women use a part of their bodies to produce milk. In doing so, the moral obligation of giving the best to our babies becomes even more salient, and the failure to do so makes us less confident in our embodied experience and more dependent on standardized health interventions (Brown, 2016; Símonardóttir and Gíslason, 2018). Unfortunately, some of the power dynamics that emerge here may interfere with the necessary process of connecting with our bodies during breastfeeding. In a study conducted by Furber and Thomson (2010), midwives admitted the language they used with the women they support was sometimes contradictory with patient-centered care, referring to them as “girls” in a very paternalistic style. Similarly, some studies have also revealed that mothers experience healthcare professionals' language as disempowering and coercive, as opposed to genuine encouragement (Graffy and Taylor, 2005). Since these communication aspects contribute to breastfeeding cessation, I argue that by focusing on one element of the dyad, not only do they misrepresent the nature of breastfeeding but they also end up threatening the very nature of what the externally regulated breastfeeding practices are trying to protect at all costs, the baby's health. Furthermore, as Van Esterik (1996) recommends, it is paramount that the welfare of both mother and child are considered and that “we must develop strategies for framing the issue in non-judgmental ways.”

My sister had a similar journey with pain, and her experience allowed me to find momentary confidence, but this was short-lived, partly because of my dependence on externally regulated practices, which kept interpreting my pain as a sign of error. After many frustrating attempts with well-intentioned though not experientially trained healthcare professionals, I found empathetic and empowering support through a not-for-profit breastfeeding awareness organization I came in touch with.

*After I came out of the hospital, I was determined to continue breastfeeding even with the pain. I wanted to experience the beauty but also to feel I had done everything in my power to provide my baby with those breastmilk benefits. Since I gave birth over the summer, I was seen by a different range of health care professionals (temporary staff), each with their very well intended breastfeeding advice. Some of them tried to “improve” the latching to see if the pain I was constantly referring to was the cause, often making it worse. Against this inconsistently qualified advice, I decided to join a breastfeeding support group run by an eco-feminist association who really supported me throughout the process. Their nurse assess the dyad and found that one of potential causes for the pain was my baby's hidden frenulum*.

Although the pain did not change much after removing the frenulum, this nurse was the first professional who validated that my pain was not only an error but also a dimension of our breastfeeding experience and that judging by my baby's development, breastfeeding was going very well. This intervention increased my confidence and allowed me to reconnect with my body, which, in turn, empowered me to stop the painful breastfeeding positioning corrections in subsequent check-ups. In short, it allowed me to become less reliant on external monitoring and more trustworthy of our self-regulated process. Hence, I argue that it is both possible and necessary to reconcile the respect for the self-regulatory nature of the process with the provision of qualified support. This way, the dyad is supported to overcome potential obstacles while the embodied and self-regulated nature of the breastfeeding process is preserved.

The WHO's recommendations are targeted at the general population to get the maximum benefits for a great majority. However, the complexity of their implementation is underestimated, and it requires investment and commitment on different levels. Evidence has shown that increasing breastfeeding rates and women's satisfaction is possible when several factors are considered. First, there is a commitment to implement evidence-based protocols continuously. Second, healthcare workers should have the resources (allocated time and adequate staffing), have been trained to understand the physiology and the emotional side of this dyadic process, and have the communication and technical skills to support mothers throughout the process in the beginning, in the middle, and in the end (Pérez-Escamilla and Hall Moran, 2016; McFadden et al., 2017). In short, the WHO's ambitious breastfeeding targets cannot be improved unless there is a strong investment in breastfeeding support in our healthcare system so that professionals are qualified and have the resources to support the wellbeing and the self-regulatory equilibrium of the dyad, as it is only through their interaction that breastfeeding benefits can be sustained.

### 3.2. The 6- to 12-month period (and beyond): A shift in public perception

Current evidence-based recommendations about breastfeeding state that breastmilk (or formula) is the main drink babies should have even with the introduction of solids at 6 months and up to the babies are 12 months of age (GPC, 2017). Despite this, I began to notice a shift in social acceptance since the introduction of solids at 6 months, but this became most evident from the moment she turned 12 months (see Table 1 for an illustration). However, the first time, I notice breastfeeding disapproval was precisely from a gynecologist when my baby was only ~3 months old.

*Gyne: “Are you still breastfeeding?”*

*Me: “Yes! I said.” I proudly explained to her all the work my baby and I had to do to get where we were, surgery involved, and found myself expecting a “pat in the back,” after all, that's what the WHO tells us to do*.

*Gyne: Oh well, not long now. When she is 3 months, you can get your freedom back*.

*Me: Oh, no. Now we will continue, minimum till she is 6 months on demand, and after that, as the WHO says ideally the first two years. I don't think I will be able to do 2 but at least one year I might do*.

*Gyne: Oh don't tell me you are one of those crazy women that wants to breastfeed for ever. Do you work?*

*Me:Oh, yes. I am a Senior Lecturer, but I am on maternity leave and will be for the rest of the year*.

*Gyne: Oh, wow your job sounds nice. Don't you realize that if you obsess with breastfeeding for a long time your professional life will be over?*

I was extremely confused. This was a doctor, and she was going against the WHO's recommendation about breastfeeding on demand. Increasingly, I began to experience explicit and indirect questioning of my breastfeeding practices more widely, some examples are described later in Table 1.

*These questions were not just from family members but also from people looking after their children in the park and even from some healthcare professionals. “How much longer are you going to do it?,” “Doesn't she do it too often?,” “Would she eat after that?,” “But, how about your work, won't this take too much of your time?” … So when I finally accepted our gains, I already seemed to be doing it wrong for many …*

Breastfeeding experiences are shaped not only by your journey but also by the meaning significant others ascribe to it, as well as the wider cultural discourses and norms. Within our immediate circle, sharing the value of breastfeeding and providing emotional and material support to partners, and in some cases, maternal mothers, seem to play a significant role in women's decisions and ability to maintain breastfeeding (Lavender et al., 2006; Rempel and Rempel, 2011; Bookhart et al., 2022). More widely, breastfeeding decisions and experiences are also influenced by the position Western society has around breastfeeding. Nowadays, this position seems to be rather ambivalent. On the one hand, considering the health benefits it attracts, society regards breastfeeding as an indicator of good mothering (Leeming et al., 2013). On the other hand, accepting breastfeeding means understanding that this may happen outdoors, yet studies suggest that breastfeeding in public spaces is still problematic. In a recent survey study conducted in Germany, about one in six respondents explicitly rejected breastfeeding in public, and acceptance had declined from the same survey conducted in 2016 (Lücke et al., 2020). According to Leeming et al. (2013), the strong sexualization of women's breasts in our society explains people's resistance to tolerating breastfeeding in public. In their qualitative diary study, the authors found that mothers had to manage the tensions between breastfeeding and the comfort of others, an act they called “socially sensitive lactation.” Similarly, in a study conducted with Australian mothers and their social networks, the authors found that there were social rules that governed what “appropriate” breastfeeding was in public, which meant that women were expected to minimize the discomfort this created for other people. Specifically, they ought to cover up not to expose their breast and protect themselves from the unwanted male gaze (Sheehan et al., 2019).

In my experience, the ambivalence was timely sequenced in that I hardly felt any strong social disapproval until the baby was ~6 months old. I then went from being the “responsible mum” to a sort of “laidback hippie,” a woman that was “too invested on breastfeeding” at the expense of forgetting about my other social roles. In this patronizing game of telling mothers how to do it, I liken society to the ambivalent parent that simultaneously asks you to take initiative while questioning and undermining your actions; a society that tells you how to do mothering, and simultaneously blames you for not deciding your path. In attachment theory terms (Bretherton, 1992), the upbringing style creates dependent, anxious, and insecure citizens. In this context, I see breastfeeding as an initiation ritual to the guilt, shame, and insecurity spirals that make us more needy of expert advice in modern motherhood (Shuterland, 2010). My way to cope with this was through performing identity work.

### 3.3. Identity work and motherhood discourses

Identity work involves “the narrative processes of self-making that parents [mothers in this case] engage in, as they raise their children” (Faircloth, 2009, p. 15). Up till my encounter with the gynecologist, I was not conscious of my identity work, as my sole focus was on crafting the technical aspects of breastfeeding. It became more of a conscious effort when the contradictions between (and within) standardized health prescriptions and the social acceptability of breastfeeding became most evident.

Sjöberg (2019) identifies two dominant, completely opposed motherhood discourses that guide mothers' identity work; the intensive motherhood discourse popularized by Hays' influential work (1996) and the modern–equal motherhood discourse. In the intensive motherhood discourse, women represent the traditional traits society identified with mothers; she is empathetic, sensitive, patient, calm, fully committed to the motherhood project, and happy to do so. According to Hays, this model rests on a series of core beliefs, that children need constant caring by their mothers, that they need experts to help them, and that they must spend all their time, money, and head-space on their children. For Hays, “there are choices about becoming a mother but not about being a good one. If you are a good mother, you must be an intensive one” (Hays, 1996). Although this could be thought to represent traditional families, Ennis (2014) stresses that today intensive motherhood applies even more so to working mothers, as they practice a more “intense” motherhood in their attempt to compensate for the time they spent at work. On the other side of the coin, modern–equal motherhood discourse draws on critical feminism and deconstructs the biological differences so that the “magical” bond between a mother and her children is not used to sacrifice women's independence (Sjöberg, 2019).

Hay's intense motherhood ideology is still present today, although it has probably evolved into risk-managing motherhood (Lee et al., 2010). The risk-managing motherhood describes mothers who need to learn about all the risks associated with each stage of baby development (starting during pregnancy or before) so that they have the best evidence they need to deal with those risks and ensure optimum baby development (Lee et al., 2010; Sjöberg, 2019). In line with Kugelmann (2003) views, we diligently put all the effort under the illusion of reaching the never-ending goal; yet in doing so, we undermine our capacities to co-define healthiness and deepen our reliance on standardized health experts. Another derivation of intense motherhood that is present nowadays is “attachment motherhood.” Faircloth (2009) identifies three key characteristics “valuing maternal-infant proximity over a long period, typically breastfeeding on cue, co-sleeping, and baby-wearing” (p. 15). Although both risk-managing and attachment motherhood are permutations of intense motherhood, I argue that a critical difference between them is attachment motherhood's resistance to depending on standardized health advice, drawing instead on the collective resources their community offers (Faircloth, 2009, 2010).

I found that my breastfeeding practices aligned with some of the characteristics of the attachment motherhood ideology, although none of it came out of a conscious decision initially but as a necessity. For instance, we initially wanted her to sleep in her own cot. However, co-sleep happened as a necessity derived from breastfeeding on demand, which for us meant every single hour for many months. As time passed, and I began interacting more with women who were in my same position, I found that engaging in identity work to conform to this motherhood ideology allowed me to counter-argue some of the contradictory breastfeeding messages from different social actors, particularly when I decided to prolong breastfeeding, and I went from responsible mum to “laidback hippie mum.” As Ennis (2014) states,

We all want to find in somewhere because it brings us security and fitting in the “good motherhood” club that society portrays is appealing to mothers where we all feel a sense of self-worth and agency, a form of denying the dominance by a patriarchal system.

Nevertheless, I had been sufficiently socialized into and compliant with the standardized health powers throughout my life to allow me to betray the attachment motherhood ideology whenever the pressures of risk-managing my baby's development were stronger than my need to fit in. In this way, I felt I was somehow navigating between risk-managing and attachment motherhood ideologies.

### 3.4. Reconciling feminist values and breastfeeding

The feminist movement has been traditionally ambivalent about breastfeeding. The most critical positionings consider that “breast is best” campaigns exaggerate the claims about breastfeeding to put women back into the private sphere and reduce motherhood to infant feeding practices (Hausman, 2013). This campaign leads to the characterization of good mothers as those who breastfeed and bad mothers as those who “choose” formula. According to Smith (2018), breastfeeding reinforces gender inequality in so far as it feeds and it is consistent with “gendered boxes” that stop women from engaging in alternative paid work opportunities. For the second-wave feminism that began around the 70s, minimizing body differences was critical to fighting the basis for gender inequality, as women's contribution to society had been reduced to the biological functions of birthing and nurturing (Smith, 2018). While their contribution to the fight for our rights was incommensurable, I find that the minimization of biological differences does a disservice to the reality of breastfeeding, the needs, and the achievements of our bodies.

The inexplicable animal tingly sensation you feel in your breast when your baby is nearby, the milk dropping inadvertently when your body senses she is near before you have actually seen her presence. Your head telling you, get out, you need to leave alone and your body firmly grounding you next to her in case she needs you…

The denial of biological differences is to be challenged as much as the reduction to mere biology, as it also contributes to deepening existing gender inequalities by failing to appropriately support the needs of lactating mothers. According to Smith (2018),

We need to challenge policies and norms that treat women as if they were no more than their biology as well as those that treat women as if they were biologically similar to men, for neither is true. Feminists and others have focused more on the former and less on the latter. But practices and norms that fail to support women's bodies in public spaces, or those that seemingly expect women to leave their bodies behind when they leave the privacy of their own homes, place further constraints on women's bodies and opportunities, thus reinforcing gender inequalities. Breastfeeding policy and practice that emerge from the vantage point of lactation as a biological process leads us to consider how we can support women's biological needs and, as lactation requires, simultaneously make it possible for mothers and babies to be together without undermining women's status and gender equality or recreating socially constructed gender inequities. (p. 298).

Third-wave feminism puts the focus on nurturing and protecting women's experiences, including breastfeeding. They acknowledge that for some women, breastfeeding is indeed an empowering experience, one that enables us to enhance our baby's development in a way that is less dependent on the externally regulated approach. As Bracken-Hull (2013) argues,

Instead of relying on expensive formulas, breastfeeding women are likely more conscious of the wonder of their own biology, thus increasing their confidence in and appreciation for their bodies (p. 2).

This confidence and appreciation is something I did not start feeling until breastfeeding was established, and admittedly, it was often shut by the contradictory messages about extended breastfeeding. Furthermore, although there were intense moments of powerful empowerment, reconciling breastfeeding and feminist values on a day-to-day basis was a challenge for me, and one that required frequent identity work. The eco-feminism positioning, which emphasizes the empowerment that comes from being able to nurture life allowed me to at least partially resolve this cognitive dissonance. As Bracken-Hull (2013) argues, “some women have decided not to surrender their rediscovered power to the historical truism that if a woman breastfeeds, she will become housebound” (p. 3).

In recognizing the contribution that breastfeeding women have made to society, and as a way to reconcile ambivalent positionings around breastfeeding, some scholars promote the provision of employment benefits and financial compensation (Bracken-Hull, 2013). As Palmer (2009) suggests,

If a multinational company developed a product that was a nutritionally balanced and delicious food ... that both prevented and treated disease, cost almost nothing to produce ... the announcement of this find would send its shares rocketing to the top of the stock market. The scientists who developed the product would win prizes .... Women have been producing such a miraculous substance ... since the beginning of human existence, yet they form the least wealthy and the least powerful half of humanity.

Breastfeeding is a highly moralized matter, mistakenly characterized as organic and natural, ergo, trouble-free and magically learned; however, it is often painful, its dyadic nature poorly understood, and it requires qualified support. Arguably, the choice to breastfeed or not, or at least to have to try it, is hardly ever just personal in a society where pressures to do so are just too strong. While we must do better to support and respect women's breastfeeding choices, we should equally advocate for improvements in the medical and social support systems they need to breastfeed if they wish to do so.

## 4. Conclusion

The portrayal of breastfeeding as trouble-free does not represent the reality of women's lived experiences. Considering the low uptake of breastfeeding in developed countries, this characterization is both ineffective and damaging for women as they keep questioning their (unable) bodies and, in turn, their motherhood, which places them in a position of deficit right from the start when their bodies are most vulnerable during the postpartum period.

Breastfeeding promotion and education should acknowledge that although it is a natural and physiological process, it often involves a learning curve, one where appropriate and consistent support must be offered. It should also feature the fact that, particularly during the early stages, difficulties are likely to be encountered—pain (and its multiple meanings), uncertainty (milk production and weight gain), and social ambivalence.

I was able to navigate these challenges, thanks to the qualified support I found in a not-for-profit breastfeeding support organization, and the identity work I performed, but I am aware that even knowing about these resources was born out of the privilege I had as an educated, middle-class woman, who could afford to take a long maternity leave and had a strong support system particularly my partner, my sister and my mum.

It is important to acknowledge that this article is mainly based on the report of a single individual, which limits the generalization of my experience and findings. However, I have minimized the impact of this limitation by comparing and contrasting my experiences to those reported by women in other studies.

To conclude, first, we must improve our breastfeeding promotion messages and validate the variety of breastfeeding experiences women have, empowering them to trust their journey. Second, while health systems and professionals should accept women's breastfeeding choices, our healthcare system needs to seriously invest in the provision of staffing and high-quality training so that professionals can adopt a compassionate, non-judgmental stance to support women, whatever feeding decision they make. Third, we need to work together to create a society that not only understands the value of breastfeeding but also that is aware and accepting of its implications. In short, unless we acknowledge the physical and social complexities of the process, and our healthcare systems make the necessary investments, breastfeeding rates will continue to suffer, and women continue to interiorize this as their failure.

## Data availability statement

The original contributions presented in the study are included in the article/supplementary material, further inquiries can be directed to the corresponding author.

## Ethics statement

Ethical review and approval was not required for the study involving human participants in accordance with the local legislation and institutional requirements. Written informed consent to participate in this study was not required from the participants in accordance with the national legislation and the institutional requirements.

## Author contributions

The author confirms being the sole contributor of this work and has approved it for publication.
